# Extended Physicochemical Characterization of the Synthetic Anticoagulant Pentasaccharide Fondaparinux Sodium by Quantitative NMR and Single Crystal X-ray Analysis

**DOI:** 10.3390/molecules22081362

**Published:** 2017-08-17

**Authors:** William de Wildt, Huub Kooijman, Carel Funke, Bülent Üstün, Afranina Leika, Maarten Lunenburg, Frans Kaspersen, Edwin Kellenbach

**Affiliations:** 1 DTS Aspen Oss B.V., 5223BB Oss, The Netherlands; wdewildt@nl.aspenpharma.com (W.d.W.); i.funke@kpnplanet.nl (C.F.); bustun@nl.aspenpharma.com (B.Ü.); aleika@nl.aspenpharma.com (A.L.); mlunenburg@nl.aspenpharma.com (M.L.); fmkaspersen@hotmail.com (F.K.); 2 Bijvoet Center for Biomolecular Research, Crystal and Structural Chemistry, Faculty of Science, Utrecht University, Padualaan 8, 3584 CH Utrecht, The Netherlands; Huub.Kooijman@shell.com

**Keywords:** Fondaparinux sodium, extended physicochemical characterization, qNMR, single crystal X-ray structure, reference standard, iduronic acid conformation, Arixtra^®^

## Abstract

Fondaparinux sodium is a synthetic pentasaccharide representing the high affinity antithrombin III binding site in heparin. It is the active pharmaceutical ingredient of the anticoagulant drug Arixtra^®^. The single crystal X-ray structure of Fondaparinux sodium is reported, unequivocally confirming both structure and absolute configuration. The iduronic acid adopts a somewhat distorted chair conformation. Due to the presence of many sulfur atoms in the highly sulfated pentasaccharide, anomalous dispersion could be applied to determine the absolute configuration. A comparison with the conformation of Fondaparinux in solution, as well as complexed with proteins is presented. The content of the solution reference standard was determined by quantitative NMR using an internal standard both in 1999 and in 2016. A comparison of the results allows the conclusion that this method shows remarkable precision over time, instrumentation and analysts.

## 1. Introduction

**Figure 1:**
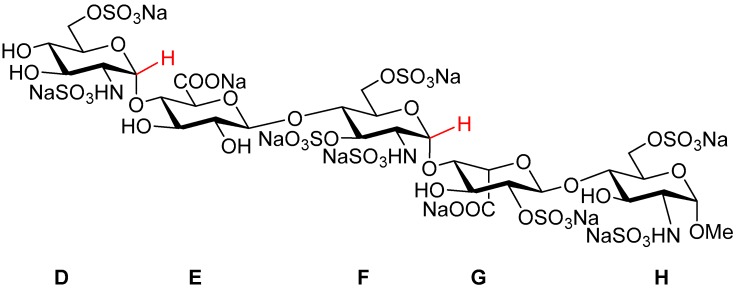
Structural formula of the synthetic anticoagulant pentasaccharide Fondaparinux sodium. The anomeric D1 and F1 protons used for qNMR integration have been indicated in red.

Fondaparinux sodium (referred to as ‘Fondaparinux’ in this article; Figure 1) is a synthetic pentasaccharide [1] derived from the high affinity antithrombin III binding site in heparin, modified by a methyl group at the reducing end. It is the active pharmaceutical ingredient of GSK’s anticoagulant drug Arixtra^®^ and a selective aXa inhibitor through antithrombin III [2] and lacks aIIa activity as a result of its low molecular weight. As part of the extended physical characterization of Fondaparinux, both structure (by X-ray) and standard content (by qNMR) are described in this paper. The X-ray structures of Fondaparinux have previously been limited to co-crystals in complex with a number of proteins [3,4,5,6,7,8]. Also, related synthetic (penta) saccharides complexed with protein have been reported [9,10]. Furthermore, uncomplexed Fondaparinux (solution) structures determined by (a combination of) theoretical calculations and NMR spectroscopy have been described [11,12,13,14,15]. In solution, ^3^*J*(^1^H, ^1^H) coupling constants allow to determine the conformation of the pyranose residues in heparin. The glucosamine and glucuronic acids residues adopt a stable ^4^C_1_-conformation. However, the iduronate ring adopts ^1^C_4_ and ^2^S_0_ conformations rapidly interconverting on the NMR time scale [11,12]. Complexation by proteins locks the iduronate residue in a single ^2^S_0_ conformation both in the crystal [5,6,9,10] and in solution [16]. Also in the crystal of uncomplexed Fondaparinux, the occurrence of a single iduronate conformation is foreseen. Here we report the X-ray single crystal structure of uncomplexed Fondaparinux and compare it to the conformation of Fondaparinux in solution, as well as complexed with proteins.

The content of ampoules of the Fondaparinux solution reference standard was determined in both 1999 and 2016 by quantitative NMR (qNMR) using an internal standard. The results of both determinations are discussed here. The ampoules of this standard are used to determine the Fondaparinux content of Fondaparinux batches. For consistency, the ability to assure the long-term stability of the standard content is crucial.

## 2. Results

**Figure 2:**
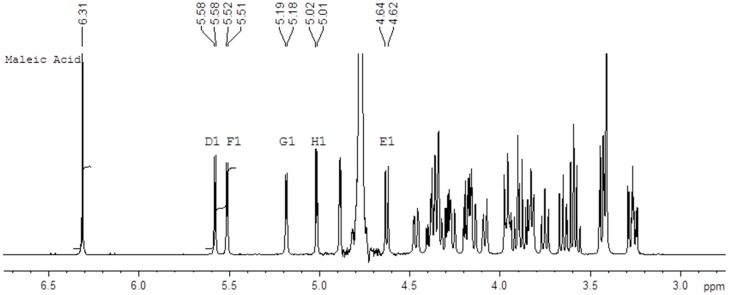
Example of the 500 MHz ^1^H-NMR spectrum of a mixture of the Fondaparinux sodium standard and maleic acid in D_2_O with the relevant integrated signals.

qNMR is a well-established and accepted method for content determination [17,18,19] and is described in major pharmacopoeia [20,21,22]. The strength of qNMR lies in the fundamental property that, under the right conditions, the integrated response of a proton in ^1^H-NMR will be identical in every molecule. This implies that a content determination can be carried out relative to a completely unrelated (but well-characterized) standard. We applied qNMR for the content determination of ampoules standard solution of the relatively complicated compound Fondaparinux using a maleic acid standard. Figure 2 shows an example of the 500 MHz ^1^H-NMR spectrum of a mixture of the Fondaparinux standard and maleic acid in D_2_O with the signals used for integration indicated. We performed these determinations on ampoules of the same standard both in 1999 and 2016 using an identical protocol which yields a unique opportunity to assess the long-term reproducibility of the qNMR method. The content determination in 1999 was performed at 400 MHz using a conventional probe whereas the content determination in 2016 was done at 500 MHz using a cryo probe. Different batches of maleic acid were used in 1996 and in 2017. For each experiment the tests were done by two different analysts and on two different days. The differences between the determinations are listed in the experimental section (Table 4).

The current analysis yields a content of 9.75 mg/mL with an SD of 0.06 mg/mL for the standard. The analysis performed in 1999 yielded a content of 9.64 mg/mL with an SD of 0.08 mg/mL. The two results are therefore not significantly different. The close correspondence between the two values obtained about 17 years apart demonstrates the intrinsic robustness of the qNMR content determination. Moreover, the differences between the two values is unlikely be due to degradation since the current content is slightly higher than the one determined 17 years ago and no signals pointing at degradation are currently detected in NMR.

### 2.1. Fondaparinux Single Crystal X-ray Structure 

**Figure 3:**
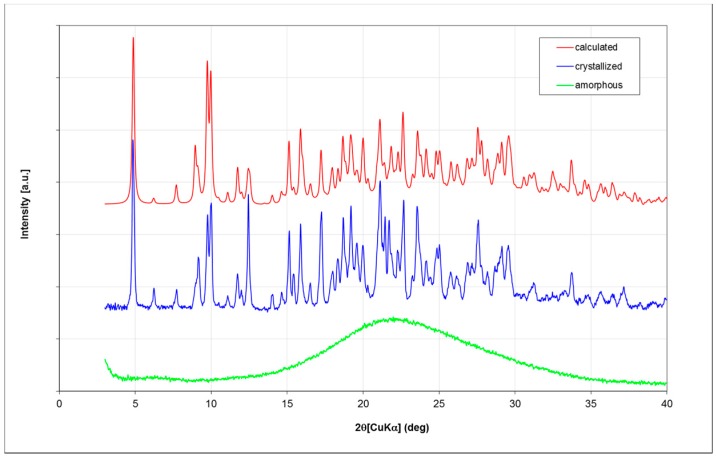
Comparison of the observed powder diffraction patterns of amorphous (green) and crystallized Fondaparinux (blue) with the pattern calculated from the single-crystal structure (red). The patterns are given an arbitrary displacement along the intensity axis to aid comparison. For the calculated pattern, the unit-cell parameters were refined using the Rietveld method to allow for the difference in data collection temperature. The reflection positions of observed and calculated patterns show a good correlation. The relative intensities show some differences, which are most likely due to the limited number of crystallites in the powder measurement, some preferred orientation effects and small changes in structure (especially in the water molecules) due to the temperature difference.

**Figure 4:**
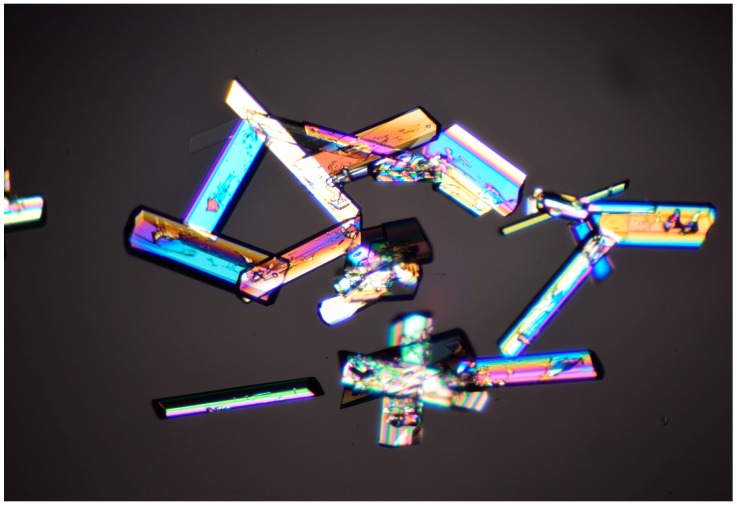
Polarized light microscopic picture at 40× magnification of Fondaparinux crystals formed from a water/ethanol solution.

Fondaparinux drug substance is industrially obtained as an amorphous powder as is evident from its X-ray powder diffractogram (XRPD; Figure 3; in green). Obtaining Fondaparinux single crystals was challenging, probably due to its inherent flexibility and linear structure. Ultimately, crystals were obtained as described in the Materials and Methods section. Several batches of crystals were produced. For a crystal from one batch, a full single-crystal structure determination was undertaken. A crystal from another batch was used for a unit-cell determination to ensure the similarity of these batches. Finally, an XRPD measurement was done for one batch of crystals see Table 7 for unit-cell comparison). In Figure 3, the blue trace shows the XRPD patterns of crystalline Fondaparinux. The XRPD pattern calculated from the crystal structure is shown as the red trace in Figure 3. A polarization microscopy image of representative crystals is displayed in Figure 4.

### 2.2. Structural Features of the Crystal Structure

**Figure 5:**
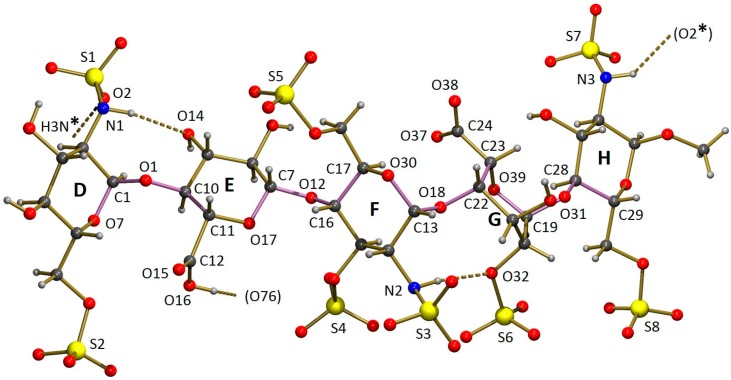
Perspective drawing of part of the asymmetric unit of the crystal structure of Fondaparinux. Sodium ions and water molecules (coordinated and non-coordinated) are excluded for clarity. Rings and atoms mentioned in the discussion are labelled. The bonds defining the glycosidic conformation are highlighted in pink. Hydrogen bonds are marked with dashed lines. N3-H3N donates a hydrogen bond to a translation related Fondaparinux molecule (*x* − 1, *y*, *z* − 1). The involved symmetry positions are indicated with a *. Hydroxyl O16 donates a hydrogen bond to a water molecule not shown.

The asymmetric unit of the crystal structure of Fondaparinux contains a pentasaccharide anion, with one of the carboxylic groups protonated (vide infra), nine sodium cations, 17 water molecules coordinated to one or more sodium atoms and 15 non-coordinated water molecules. A perspective drawing of the molecular structure of the pentasaccharide Fondaparinux, along with the atomic labeling scheme of the non-hydrogen atoms and the labeling scheme of the rings is given in Figure 5.

The sodium ions are coordinated by numerous oxygen atoms from the pentasaccharide and extensively co-crystallized water molecules. The seven ordered sodium ions are coordinated by six oxygen atoms each. For the ordered ions, most Na...O distances fall in the range 2.3–2.7 Å. However, a small number of Na...O distances up to 2.9 Å are observed. The two disordered sodium ions are also surrounded by a total of six oxygen atoms that can be assigned to the coordination shell, but this shell is somewhat larger than that observed for the ordered sodium atoms. As a result of this, the sodium can take up several positions and some of the individual disorder components are only directly coordinated by five oxygen atoms. Full details of the observed coordination distances are given in the deposited Crystallographic Information File. Through these coordinative bonds an infinite three-dimensional structure is formed. A perspective drawing of the infinite substructure formed by sodium counter-ions, sulfate groups and coordinated water molecules is included in the Supporting Information. The final model contained no solvent accessible void. DFT calculations also indicated extensive coordination to both sodium ions and water (e.g., [13,15]).

## 3. Discussion

The individual glucosamine (D, F and H) rings have adopted the conventional stable ^4^C_1_ chair conformation. The glucuronic acid ring (E) is in a slightly distorted ^4^C_1_ chair-conformation. The iduronic acid G ring conformation has been the subject of intense debate [11,12,13,14,15,23,24,25,26]. Uncomplexed in solution, its structure cannot be described by a single conformer but is an equilibrium of ^1^C_4_, chair and ^2^S_0_. Skew-boat conformations with the equilibrium shifted to the ^2^S_0_ conformation [11,12,13,14,15,23,24,25,26]. The conformation is ^2^S_0_ in complex with antithrombin III [5,6,16].

**Figure 6:**
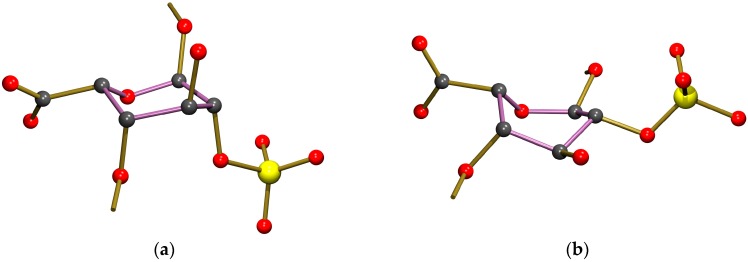
A comparison of the conformations of the iduronic acid G ring structures in uncomplexed Fondaparinux (**a**) and Fondaparinux co-crystallized with antithrombin (**b**); from PDB entry 2GD4) [5].

In our crystal structure, the iduronic acid ring (G) is in a chair conformation, heavily distorted towards a half-chair conformation with the best-fitting local twofold rotation axis running through the midpoint of the bond ring O–C5 (crystal structure numbering O39–C23; see Table 1 for quantification using Cremer & Pople puckering parameters [27]). The iduronic ring conformation is therefore clearly different from the conformation reported by Johnson et al. [5], who found a skew-boat conformation (related Cremer & Pople puckering parameters are included in Table 1). A comparison of the conformations of the iduronic acid G ring in both structures is given in Figure 6. Interestingly, in the co-crystal of heparin and teichoic acid α-glycosyl transferase the iduronic acid adopts a chair conformation according to the coordinates reported by Sobhanifar et al. ([8], PDB entry 4X7R), related Cremer & Pople puckering parameters are also included in Table 1.

The overall conformation of the pentasaccharide can be described in terms of the torsion angles of the glycosidic links between the carbohydrate rings. An alternative description uses the dihedral angles between least-squares planes fitted through the ring atoms. Numerical data for both descriptions are given in Table 2. Table 2 contains the same set of descriptors for the pentasaccharide molecules co-crystallized with antithrombin S195A, platelet factor 4 and a glycosyl transerase as well as for the structure in solution determined by NMR. A comparison of the free and co-crystallized pentasaccharide shows only relatively small differences when the glycosidic links between adjacent sugar rings are considered. In most cases the range of torsion angles observed is approximately 30 °C. However, in combination with the conformational change of the iduronic acid, these differences add up to a substantial difference for the overall conformation of the pentasaccharide. This can be convincingly illustrated by the angle between the least-squares planes fitted through rings D and H, which shows a difference of almost 60 °C. This difference in dihedral angles (which have a defined range of 0–90 °C) corresponds to the mean planes of rings D and H being almost parallel in the crystal structure of Fondaparinux while they are almost perpendicular in complex 2GD4.

The intramolecular hydrogen bond between rings D and E, found in the Fondaparinux crystal structure, is also present in the co-crystal with antithrombin. In the latter, an even shorter donor-acceptor distance of 2.98 Å is found, suggesting a reasonably strong hydrogen bond. The intramolecular hydrogen bond between residues F and G (iduronic acid), found in the single-crystal structure of Fondaparinux, is not present in the pentasaccharide unit in the co-crystal. The conformational change of the iduronic acid to a twist-boat has moved the accepting sulfate out of reach of the sulfamido group. In the co-crystal, this sulfamido group rotated around the bond to which it is attached to the sugar ring so that an intramolecular hydrogen bond to the neighbouring sulfate group attached to the same sugar ring can be formed (donor-acceptor is 2.98 Å, also suggesting the presence of a reasonably strong intramolecular hydrogen bond).

The sulfamido groups play an important role in stabilizing the pentasaccharide molecule in a relatively linear conformation by forming two N-H...O intramolecular hydrogen bonds. The NH hydrogen of the sulfamido group in carbohydrate residue F is involved in an interresidue hydrogen bond to an oxygen of the 2-sulfate group residue G, as shown in Figure 5. This hydrogen bond is also found in solution [15]. Previously, in solution, an intraresidue hydrogen bond between the NH hydrogen of the sulfamido group in carbohydrate residue F and the adjacent 3-sulfo group [15,28] was found. This hydrogen bond is not observed in the crystal, possibly as a consequence of the different conformation of the iduronic acid G discussed above. Geometric details are given in Table 3.

Besides the items listed in Table 3, the crystal structure displays numerous hydrogen bonds, which further strengthen the infinite three-dimensional network formed by the pentasaccharide ions, sodium ions and water molecules. As can be expected in view of their acidic nature, all SO_3_H groups are deprotonated, which is supported by the observed equality of the S–O bond lengths. 

Interestingly, the geometry of the carboxylic acid group bonded to ring E strongly suggests a protonated, neutral acid group (*d*(C12–O15) = 1.09(2) Å, *d*(C12–O16) = 1.40(3) Å), in spite of the fact that the crystallization was carried out at neutral pH. Due to high anisotropic displacement parameters, the C–O distances appear shortened. The anisotropicity is also reflected by the accuracy of these bond lengths, which is significantly lower than that of similar bond lengths in other sections of the pentasaccharide anion. The presence of a hydrogen bond acceptor at hydrogen bonding distance from O16 (see Table 3) supports the model of a protonated acid group. However, the unfavorable value of the *D*–H^…^*A* angle (131 deg) does not suggest this is a particularly strong hydrogen bond. 

The acid group bonded to ring G (iduronic acid) is deprotonated as expected, as is clearly shown by the similar lengths of the C–O bonds: *d*(C24–O37) = 1.242(11) Å and *d*(C24–O38) = 1.267(10) Å.

Based on the covalent angles between their substituents, the nitrogen atoms N1, N2 and N3 can be considered *sp*^3^ hybridized, which is common for this type of group. The coordinates of the hydrogen atoms bonded to the nitrogen atoms were derived from the availability of hydrogen bond acceptors at reasonable distances from the nitrogen donor atoms (see “Crystal Structure Determination and Refinement”).

### Absolute Configuration

The Flack *x-*parameter and its standard uncertainty *u*(*x*) can be used to assess absolute structure and absolute configuration [29,30]. A structure determination is considered to have strong inversion-distinguishing power if *u*(*x*) < 0.04. If the enantiopurity of a sample is certain, a less strict criterion is applied. When *u*(*x*) < 0.08, the experiment is considered to have enantiopure sufficient inversion-distinguishing power. If one of these criteria is met, a numerical value of *x* that lies within statistical fluctuation of zero, i.e., |*x*| < 2 *u*(*x*), assures a valid absolute structure (and absolute configuration) determination from a single crystal which is not twinned by inversion. The cited criteria are taken from Flack and Bernardinelli [31].

In this study, a value of −0.02(11) was found by classical fit to all intensities, which does not satisfy the criterion for enantiopure sufficient inversion-distinguishing power. However, as argued by Parsons et al. [32] anomalous dispersion differences can be obscured by other effects, such as absorption and extinction. If these effects are filtered out as proposed by these authors, and *x*-value of 0.017(14) is obtained, we can classify the current structure determination as having strong inversion-distinguishing power. 

As a final alternative to assess the absolute configuration, the method of Hooft et al. [33] was considered. Using Bayesian statistics, these authors offer a method that gives reliable absolute structure determinations even in cases where only weak anomalous scatterers are present. According to this method, the probability that the absolute configuration was determined correctly is calculated to be 1.00, based on the assumption of an enantiopure sample. In case the measured crystal is a racemic twin, the twin ratio, expressed as the *y*-parameter, comparable to Flack’s *x*-parameter, amounts to −0.002(14), further confirming the correct assignment of the absolute configuration of the enantiopure sample. 

## 4. Materials and Methods

### 4.1. General Information

#### 4.1.1. Materials

The Fondaparinux standard was prepared from a commercially manufactured Fondaparinux batch and underwent an additional ion exchange chromatography purification. Maleic acid batches were obtained from Acros (Geel, Belgium; in 1999) or Fluka (St. Louis, MO, USA; Lot BCBV2002V, in 2016) and used as such. The content of the maleic acid batches of 99.61 (1999) and 99. 99% (2016) was taken from the Certificate of Analysis from the supplier. D_2_O was used as a solvent since both Fondaparinux and the standard maleic acid are charged molecules and well soluble in water. 0.01% *w*/*v* TSP-*d*_4_ was used as a chemical shift reference at 0 ppm. The 500 MHz 1D ^1^H spectrum of Fondaparinux and maleic acid is displayed in Figure 1 including the D1, F1 and maleic acid proton integrals used for content determination. 

From an ampoule of Fondaparinux standard solution, 0.5 mL was transferred into an Eppendorf and 0.5 mL of a 0.67 mg/mL maleic acid standard solution was added. After stirring, the content of the Eppendorf was then frozen in a dry-ice ethanol mixture and subsequently lyophilized (>8 h, −50 °C, <300 mbar). After lyophilizing 0.7 mL D_2_O was added to the freeze-dried material and subsequently stirred until complete dissolution. The solution was then transferred into a NMR tube. Five determinations were performed by two different analysts on two different days.

#### 4.1.2. Fondaparinux Crystallization 

Fondaparinux (100 mg) was dissolved in 2.5 mL of demineralized water in a flat-bottomed flask or bottle. The temperature was increased to 70 °C to dissolve the material. 5.5 mL of ethanol at room temperature was slowly added while maintaining the temperature at 70 °C to saturate the solution (the solution became slightly turbid). The solution (or even slightly turbid mixture) was then cooled to RT. No stirring was applied.

The turbidity of the mixture increased under cooling. Over time, the turbidity decreased, and in turn, an oily liquid (or drops) was formed on the bottom of the flask. After a while, the oily drops changed and crystallized (this procedure was not an optimized process and it took weeks before crystallization occurred). Various crystallization experiments of Fondaparinux (maximum concentration 16 mg/mL) were successful with an ethanol content between 70% and 90%. In addition, when available, seeding crystals were used to speed up the crystallization process.

### 4.2. Methods

#### 4.2.1. qNMR

##### Spectrum Acquisition and Processing

^1^H-NMR spectra were recorded on a Bruker NMR spectrometer (Bruker BioSpin, Billerica, MA, USA) according to the settings in Table 4.

The recorded spectra were manually phased and baseline corrected, and the signals of maleic acid and the anomeric protons D1 and F1 (indicated in red in Figure 1) were integrated as described in the protocol. The content of Fondaparinux in an ampoule was then calculated according to the formula: C_FP_ = W_MA_/V_FP_ × I_FP_/I_MA_ × MW_FP_/MW_MA_ × P_MA_/P_FP_ where C_FP_ = concentration of Fondaparinux in the ampoule, W_MA_ = weight of maleic acid (=concentration stock solution (mg/mL) × 0.5 mL); V_FP_ = volume of Fondaparinux taken from the ampoule (typically 0.5 mL); I_FP_ = integral of selected signals of Fondaparinux; I_MA_ = integral of maleic acid set at 2.0000 for each sample MW_FP_ = molecular weight of Fondaparinux (1728.088); MW_MA_ = molecular weight of maleic acid (116.07); P_MA_ = purity of maleic acid (99.99%); P_FP_ = purity of Fondaparinux (assumed to be 100%). The determination was performed in fivefold by two technicians on two different days and the average Fondaparinux content (mg/mL) was reported including the standard deviation and the relative standard deviation (RSD).

### 4.3. XRPD

X-ray powder diffractograms were obtained on a Miniflex600 diffractometer (Rikagu, Tokyo, Japan) using the settings listed in Table 5 below.

### 4.4. Crystal Structure Determination and Refinement 

A colorless, block-shaped crystal of approximate dimensions 0.10 × 0.20 × 0.20 cm was fixed to the tip of a glass capillary and transferred into the cold nitrogen stream on a kappa-CCD diffractometer (Bruker-Nonius, Billerica, MA, USA) on rotating anode. Raw data were reduced with DENZO [34]. Crystal data and details on data collection and refinement are presented in Table 6. 

The unit-cell parameters were checked for the presence of higher lattice symmetry [35]. Data were not corrected for absorption. The structure was solved by automated direct methods (SHELXS86 [36]). Refinement on *F*^2^ was carried out by full-matrix, least-squares techniques (SHELXL 2016-6 [37]); no observance criterion was applied during refinement. Two of the sodium ions and some of the water molecules coordinated with one of these sodium ions were found to be disordered. A disorder model with two positions for each of the atoms involved was introduced. The two sites for each sodium atom are close to each other. The site occupation factor of the major component refined to 0.502(11) for Na7 and 0.549(17) for Na9 and associated disordered water molecules. All hydrogen atoms were included in the refinement on calculated positions riding on their carrier atoms. The methyl hydrogen atoms and the hydroxyl hydrogen atoms were refined as rigid groups, allowing for rotation around the C–O bonds. Starting positions of the methyl hydrogen were derived from a difference Fourier map. The hydrogen atoms of the non-coordinated water solvent molecules could not be located on difference Fourier maps. Furthermore, the coordinates of these atoms could not be derived unambiguously from the distribution of potential hydrogen bond donors and acceptors. Most likely, there is extensive disorder in the hydrogen atoms of the non-coordinated water molecules. Displacement parameters of the oxygen atoms of the non-coordinated water molecules indicate that there is a slight disorder in their positions as well. Therefore, no further attempts were made to include these hydrogen atoms in the atomic model. All ordered non-hydrogen atoms were refined with anisotropic atomic displacement parameters. Atoms in the major disorder component were refined with isotropic displacement parameters, which were coupled to the isotropic displacement parameters of the related atoms in the minor component. Hydrogen atoms were refined with fixed isotropic displacement parameters related to the value of the equivalent isotropic displacement parameters of their carrier atoms by a factor of 1.5 for methyl, hydroxyl and water hydrogen atoms, and 1.2 for all other hydrogen atoms.

The Flack *x*-parameter [29], derived during the final structure-factor calculation, amounts to −0.02(11) by classical fit to all intensities and 0.017(14) from 7033 selected quotients (Parsons’ method, [32]). Refinement of the inverse absolute structure resulted in an *x*-parameter of 1.04(11) (value derived during final structure-factor calculation). Figures of merit for this inverted structure are *R*1 = 0.0791, *wR*2 = 0.2223 (weighting scheme not optimized) and *S* = 1.0. Refinement of a racemic twin model gave a twin ratio of reported versus inverted structure of 1.00(11):0.00; the twin model was therefore not used. 

Neutral atom scattering factors and anomalous dispersion corrections were taken from the International Tables for Crystallography [38]. Geometrical calculations and illustration were performed with PLATON [39].

#### Unit Cell Determination

At *T* = 150 K, a data set was collected, consisting of a scan of 20 deg of ϕ divided into 20 image frames, using Mo*K*α-radiation (λ = 0.71073 Å). The unit cell was determined using DENZO [28]. Results are summarized in Table 7.

## 5. Conclusions

Both Fondaparinux structure (X-ray) and content (qNMR) have been addressed in this paper. The content of ampoules of a Fondaparinux solution standard was determined using qNMR. For consistency, the ability to assess the long-term stability of the content of the standard is crucial since the standard content is used to determine the content of Fondaparinux batches. The 2016 analysis yielded a content of 9.75 mg/mL (SD 0.06 mg/mL) whereas the analysis performed in 1999 yielded a content of 9.64 mg/mL (SD of 0.08 mg/mL). qNMR shows remarkable precision over time, instrumentation and analysts, pivotal for consistent content determination.

The determination of the single crystal X-ray structure including the absolute configuration of Fondaparinux crystals allowed an unequivocal proof of structure. The iduronic acid residue in the uncomplexed Fondaparinux adopts a chair conformation heavily distorted towards a half chair. Interestingly, this iduronic acid conformation was also found in the complex of heparin with wall teichoic acid transferase according to the coordinates published [8]. The iduronic acid conformation also provides solid evidence of the thermodynamic accessibility of this chair conformation of iduronic acid within a pentasaccharide, (and thereby helps to extend our knowledge of the available conformational space).

## Figures and Tables

**Table 1:** Cremer & Pople puckering parameters for the rings in the Fondaparinux structure published here (FP). Parameters for a selection of ideal conformations are included for comparison in a separate section; *k* is an integer number [27]. For comparison, the values calculated from the published coordinates of a selection of co-crystals of proteins and heparin. These are indicated with their PDB codes: 2GD4 is antithrombin S195A with heparin [5]; 4R9W is platelet factor 4 with heparin [7], 4X7R is wall teichoic acid glycosyl transferase [8]. The last column reports the values calculated from the coordinates of a solution NMR study [28]. 2GD4 and 4X7R contain two crystallographically independent heparin molecules; values for both are included.

*Reference Values*														
			θ						ϕ					
Chair			0 or 180						(any value)					
Half-chair			50.8 or 129.2						*k* × 60 + 30					
Boat			90						*k* × 60					
Skew-boat			90						*k* × 60 + 30					
***Observed Values***														
	**FP**		**2GD4 1**		**2GD4 2**		**4R9W**		**4X7R 1**		**4X7R 2**		**NMR Solution**	
	**θ**	**ϕ**	**θ**	**ϕ**	**θ**	**ϕ**	**θ**	**ϕ**	**θ**	**ϕ**	**θ**	**ϕ**	**θ**	**ϕ**
**D**	0	349	7	64	7	65	12	308	7	0	6	342	4	26
**E**	8	23	3	94	3	92	18	40	8	339	1	350	5	22
**F**	0	227	7	76	7	76	7	7	5	21	4	18	7	107
**G**	165	135	91	140	91	140	90	1	169	142	170	109	92	133
**H**	4	298	7	268	7	268	89	208	5	28	14	68	10	324

**Table 2:** Description of the overall conformation of the pentasaccharide in terms of torsion angles (deg.) of the C–O backbone in the glycosidic links (see also Figure 5 where these backbones are highlighted) and dihedral angles (deg.) between least-squares planes fitted through the ring atoms. Atom and ring labels are given in Figure 5; s.u.’s are included in parentheses. For comparison, the values for various other structures are included in the Table, see the legend of Figure 1 for details of the abbreviations used here.

Torsion Angles (deg)	FP	2GD4 1	2GD4 2	4R9W	4X7R 1	4X7R 2	NMR Solution
τ(O7–C1–O1–C10)	90.0(9)	101.4	101.4	69.5	96.4	95.5	76.9
τ(C1–O1–C10–C11)	−143.9(10)	−157.6	−158.0	−155.4	−128.2	−126.1	−151.4
τ(O17–C7–O12–C16)	−82.3(7)	−84.4	−84.3	−70.2	−94.6	−93.0	−55.2
τ(C7–O12–C16–C17)	−103.4(6)	−120.8	−121.0	−105.1	−100.5	−97.2	−111.0
τ(O30–C13–O18–C22)	88.5(7)	63.1	62.4	71.0	73.2	73.7	89.2
τ(C13–O18–C22–C23)	−145.9(6)	−157.0	−156.2	−132.0	−152.0	−146.2	−143.8
τ(O39–C19–O31–C28)	−72.0(7)	−67.8	−-67.5	−77.0	−65.2	−66.6	−73.0
τ(C19–O31–C28–C29)	−118.7(6)	−108.5	−108.7	−139.2	−106.8	−110.2	−134.0
**Dihedral Angles (deg)**	**FP**	**2GD4 1**	**2GD4 2**	**4R9W**	**4X7R 1**	**4X7R 2**	**NMR Solution**
χ (D,E)	57.4(5)	57.4	57.6	63.1	48.9	44.6	58.2
χ (D,H)	14.0(3)	71.3	71.4	31.7	13.6	12.7	30.3
χ (E,F)	57.3(5)	35.7	35.5	68.9	48.4	50.2	69.3
χ (F,G)	66.6(4)	78.8	78.8	89.7	69.7	72.2	84.0
χ (G,H)	39.3(4)	33.8	33.7	20.7	57.0	53.4	3.4

**Table 3:** Geometric details of selected hydrogen bonds (Å, deg.). Standard uncertainties are given in parentheses (only available for *D*...*A* since H atoms were not freely refined). Hydrogen bond type refers to intramolecular or intermolecular hydrogen bonds. The listed hydrogen bonds are also indicated in Figure 5.

Involved Atoms	Type	*D*–H	H^…^*A*	*D*^…^*A*	*D*–H^…^*A*
N1–H1N^…^O14	intra	0.92	2.52	3.282(14)	141
N2–H2N^…^O32	intra	0.92	2.18	3.095(9)	172
N3–H3N^…^O2 (*x* − 1, *y*, *z* − 1)	inter	0.92	2.26	2.972(12)	134
O16–H16H^…^O76 (water)	inter	0.84	1.91	2.54(3)	131

**Table 4:** Acquisition and processing settings of the qNMR experiment.

Acquisition	1999	2016
Spectral frequency	400 MHz	500 MHz
Probe	5 mm QNP probe	TCI Cryo Probe
Temperature	25 °C/298 K	
Spinning	20 Hz	Off
Flip angle	60°	
Relaxation delay	30 s	
Dummy scans	4	2
Number of scans	128	32
Number of data points	64 k	
Processing		
Exponential line-broadening	0.5 Hz	0.3 Hz
Zero-Filling	64 k	132 k

**Table 5:** XRPD settings.

XRPD Settings	
X-ray	40 kV, 15 mA
Goniometer	MiniFlex 600
Wavelength	Cu K_α_ (1.541 Å)
Filter	K_β_(Ni)
Scan speed/Duration time	2.0000 deg/min
Step width	0.0200 deg
Scan axis	θ/2-θ
Scan range	3.0000–40.0000 °C ambient
Temperature	Ambient

**Table 6:** Crystallographic data. Unit cell parameters are listed in Table 5.

Crystal Data	
Formula	[C_31_H_44_N_3_O_49_S_8_]9^−^·9Na^+^·32H_2_O
Molecular weight	2282.60
Crystal system	monoclinic
Space group	*P*2_1_ (No.4)
*D*_calc_, g cm^−3^	1.784
*Z*	2
*F*(000)	2380
µ (Mo*K*α), mm^−1^	0.401
Crystal size	0.2 × 0.3 × 0.3
Data collection	
*T*, K	150
θ_min_, θ_max_, deg	1.00, 27.49
Wavelength (Mo*K*α), Å	0.71073 (graphite monochromated)
Distance crystal to detector, mm	45
X-ray exposure time, h	7.0
Refined mosaicity, deg	0.914(1)
Data set (hkl-range)	−13:13, −29:29, −24:24
Completeness at sin θ/λ = 0.6 Å^−1^	100.0% (no refl. missing)
Total data	81362 (*R*_σ_ = 0.0498)
Total unique data	19366 (*R*_int_ = 0.0396)
Refinement	
No. of refined parameters	1114
*wR*2	0.2211
*R*	0.0786 [for 16915 *F*_o_ > 4σ(*F*_o_)]
*S*	1.041
*w*^−1^	σ^2^(*F*^2^) + (0.1328*P*)^2^ + 11.21*P*
(Δ/σ)_av_, (Δ/σ)_max_	<0.0001, 0.0002
Δρ_min_, Δρ_max_, e Å^−3^	−0.84, 1.87 (near Na)

**Table 7:** Unit cell parameters of Fondaparinux determined for the single-crystal study, the cell check of a second batch, and the powder diffraction study. In all cases systematic absences indicate the space group is *P*2_1_. s.u.’s are included in parentheses.

	Single Crystal (DW1623B)	Cell Check (DW1637A)	Powder
*a*, Å	10.0296(2)	10.0132(18)	10.144(3)
*b*, Å	23.0353(6)	23.067(5)	22.992(5)
*c*, Å	18.9666(7)	18.820(3)	18.626(6)
β, deg	104.1420(14)	103.731(2)	102.65(2)
*V*, Å^3^	4249.1(2)	4222.6(2)	4238.8(4)
Refined mosaicity, deg	0.914(1)	1.626(9)	-
*T*, K	150	150	ambient

## Supplementary Materials

Supplementary materials are available online.CCDC 1553209 contains the supplementary crystallographic data for this paper. These data can be obtained free of charge via http://www.ccdc.cam.ac.uk/conts/retrieving.html (or from the CCDC, 12 Union Road, Cambridge CB2 1EZ, UK; Fax: +44 1223 336033; E-mail: deposit@ccdc.cam.ac.uk).
