# Supplementary material for: Extended Physicochemical Characterization of the Synthetic Anticoagulant Pentasaccharide Fondaparinux Sodium by Quantitative NMR and Single Crystal X-ray Analysis
Source: Molecules. 2017 Aug 17;22(8):1362. doi: 10.3390/molecules22081362 (PMC6152090; doi:10.3390/molecules22081362)
Supplement: Supplementary file 1 [file molecules-22-01362-s001.pdf]

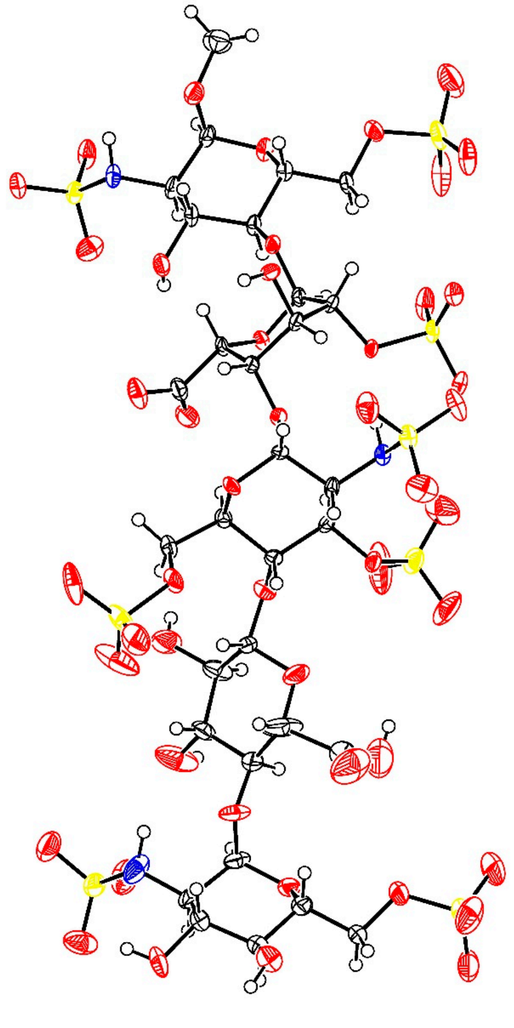

Figure S1: Displacement ellipsoid plot of the pentasaccharide molecule of Fondaparinux drawn at 50% probability level.

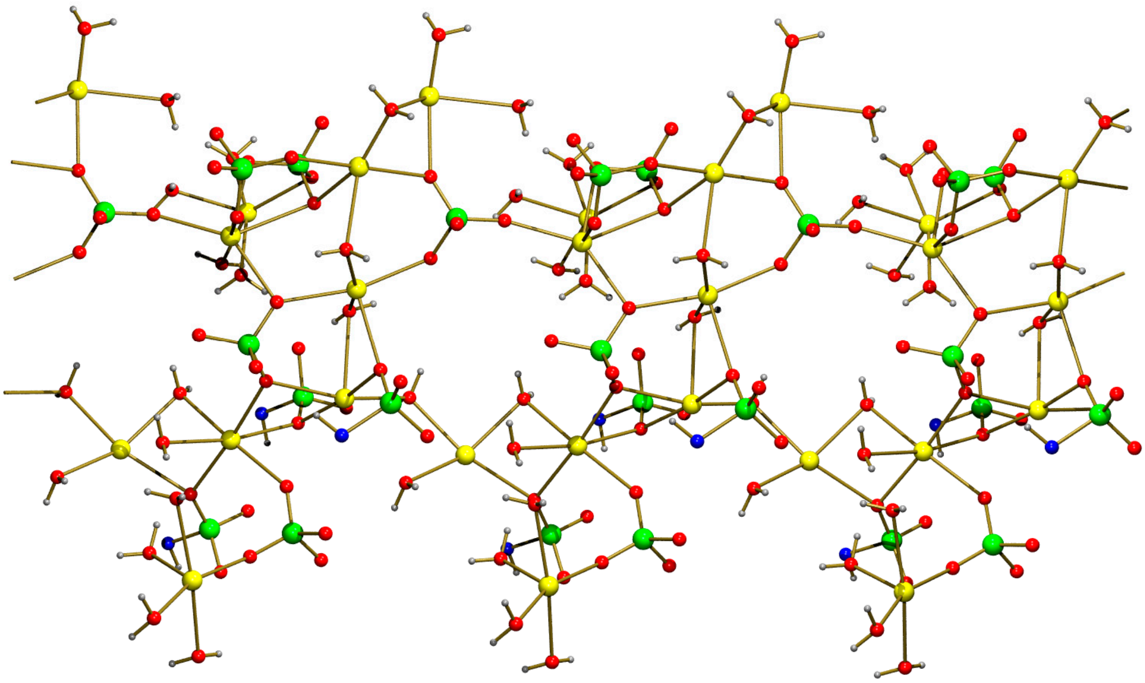

Figure S2: Impression of the crystal packing of Fondaparinux, highlighting the infinite three-dimensional framework formed by Na...O coordination in the substructure of sulfate groups, sodium and coordinated water molecules. The sugar rings have been omitted for clarity.

Table S1: Geometric details of all unique potential hydrogen bonds.

Geometric details of Potential Hydrogen Bonds; distances in Å, angles in °. Standard uncertainties are given in parentheses. Note: no standard uncertainties are given for quantities calculated from coordinates at calculated positions. Geometric criteria used for inclusion in the list:
d(D…A) < R(D)+R(A)+0.50 Å
d(H…A) < R(H)+R(A)-0.12 Å
D-H…A > 100.0°
(D = donor, A = Acceptor, R = Van der Waals radius)
ARU = Asymmetric Residue Unit, the code given is written out in equivalent positions below the H-bond table. For potential bifurcated H-bonds, the Acceptor…Hydrogen…Acceptor angle is included in the Tables, as well as the sum of all angles involving the central hydrogen, which is expected to be 360° for a truly bifurcated system.

Nr	Type	Donor-H…Acceptor	ARU	D-H	H…A	D…A	D-H…A	A…H…A*	Sum
1	Intra	N(1) -H(1N) …O(1)	[ ]	0.92	2.29	2.769(11)	112		
2	Intra	N(1) -H(1N) …O(14)	[ ]	0.92	2.52	3.282(14)	141'	73'	326
3	Intra	N(2) -H(2N) …O(18)	[ ]	0.92	2.39	2.830(8)	109		
4	Intra	N(2) -H(2N) …O(32)	[ ]	0.92	2.18	3.095(9)	172'	78'	359
5	Inter	N(3) -H(3N) …O(2)	[1454]	0.92	2.26	2.972(12)	134		
6	Intra	N(3) -H(3N) …O(40)	[ ]	0.92	2.34	2.826(9)	112'	102'	348
7	Inter	O(6) -H(6H) …O(42)	[1756]	0.84	2.11	2.819(10)	142		
8	Inter	O(13) -H(13H)…O(61)	[ ]	0.84	1.88	2.701(13)	164		
9	Inter	O(14) -H(14H)…O(71)	[ ]	0.84	2.26	2.766(18)	119		
10	Inter	O(16) -H(16H)…O(76)	[ ]	0.84	1.91	2.54(3)	131		
11	Inter	O(36) -H(36H)…O(42)	[1655]	0.84	1.95	2.773(7)	165		
12	Inter	O(44) -H(44H)…O(52)	[1455]	0.84	2.11	2.900(9)	156		
13	Inter	O(50) -H(50S)…O(44)	[1656]	0.84	2.08	2.845(8)	152		
14	Inter	O(50) -H(50T)…O(70)	[1656]	0.84	1.94	2.766(9)	169		
15	Inter	O(51) -H(51S)…O(20)	[2756]	0.84	2.51	2.796(14)	101		
16	Inter	O(51) -H(51T)…O(60)	[2656]	0.82	2.32	3.141(15)	180		
17	Inter	O(52) -H(52S)…O(19)	[ ]	0.84	2.15	2.947(10)	159		
18	Inter	O(52) -H(52T)…O(37)	[1655]	0.83	2.28	2.839(10)	124		
19	Inter	O(52) -H(52T)…O(39)	[1655]	0.83	2.42	3.093(9)	138'	66'	328
20	Inter	O(53) -H(53S)…O(37)	[1655]	0.85	1.98	2.676(10)	138		
21	Inter	O(53) -H(53T)…O(29)	[ ]	0.84	2.54	3.050(11)	120		
22	Inter	O(53) -H(53T)…O(47)	[2655]	0.84	2.23	2.964(10)	147'	85'	352
23	Inter	O(54) -H(54S)…O(73)	[ ]	0.84	2.25	2.850(17)	128		
24	Inter	O(54) -H(54T)…O(60)	[1655]	0.84	2.60	3.007(12)	111		
25	Inter	O(54) -H(54T)…O(61)	[1655]	0.84	2.41	3.199(13)	156'	90'	357
26	Inter	O(55) -H(55S)…O(43)	[1655]	0.84	2.57	3.227(15)	136		
27	Inter	O(55) -H(55S)…O(58)	[2655]	0.84	2.59	3.355(16)	152'	70'	358
28	Inter	O(55) -H(55T)…O(70)	[1655]	0.84	2.05	2.765(15)	142		
29	Inter	O(56) -H(56S)…O(42)	[1756]	0.84	2.12	2.884(10)	151		
30	Inter	O(56) -H(56T)…O(38)	[1656]	0.84	2.22	2.970(11)	149		
31	Inter	O(57) -H(57S)…O(20)	[2756]	0.84	2.16	2.784(15)	131		
32	Inter	O(57) -H(57T)…O(79)	[2756]	0.84	2.48	3.11(4)	132		
33	Inter	O(58) -H(58S)…O(38)	[2645]	0.84	1.92	2.753(11)	168		

34	Inter	O(58) -H(58T)…O(43)	[2545]	0.82	2.12	2.941(11)	180		
35	Inter	O(59) -H(59S)…O(49)	[1655]	0.84	1.93	2.762(10)	170		
36	Inter	O(59) -H(59T)…O(10)	[1454]	0.84	2.52	3.187(12)	137		
37	Inter	O(60) -H(60S)…O(37)	[ ]	0.84	2.01	2.816(12)	160		
38	Inter	O(60) -H(60T)…O(54)	[1455]	0.84	2.40	3.007(12)	129		
39	Inter	O(61) -H(61S)…O(75)	[ ]	0.84	2.00	2.81(2)	159		
40	Inter	O(61) -H(61T)…O(53)	[1455]	0.84	1.97	2.769(13)	158		
41	Inter	O(62) -H(62S)…O(76)	[ ]	0.84	1.99	2.81(3)	165		
42	Inter	O(62) -H(62S)…O(92)	[ ]	0.84	1.82	2.26(5)	111'	82'	358
43	Inter	O(62) -H(62T)…O(74)	[ ]	0.84	1.97	2.72(3)	148		
44	Inter	O(63) -H(63A)…O(73)	[1455]	0.84	1.97	2.77(2)	159		
45	Inter	O(63) -H(63C)…O(51)	[2646]	0.84	2.37	3.18(3)	159		
46	Inter	O(64A)-H(64S)…O(78)	[2656]	0.84	1.90	2.66(5)	150		
47	Inter	O(64A)-H(64T)…O(72)	[2656]	0.85	2.21	3.04(5)	166		
48	Inter	O(65A)-H(65S)…O(29)	[1455]	0.84	2.37	3.00(2)	133		
49	Inter	O(65A)-H(65T)…O(11)	[2656]	0.84	2.20	2.90(2)	141		
50	Inter	O(66A)-H(66S)…O(77)	[2656]	0.84	1.74	2.56(6)	166		
51	Inter	O(66A)-H(66T)…O(4)	[ ]	0.85	2.59	2.87(6)	100		
52	Inter	O(66A)-H(66T)…O(14)	[ ]	0.85	2.16	2.95(5)	154'	105'	359

ARU-Code expressed in equivalent positions

[1454] = -1+x,y,-1+z
[1455] = -1+x,y,z
[1655] = 1+x,y,z
[1656] = 1+x,y,1+z
[1756] = 2+x,y,1+z
[2545] = -x,-1/2+y,-z
[2646] = 1-x,-1/2+y,1-z
[2656] = 1-x,1/2+y,1-z
[2756] = 2-x,1/2+y,1-z
[2655] = 1-x,1/2+y,-z
[2645] = 1-x,-1/2+y,-z
